# MicroRNA-29 family expression and its relation to antiviral immune response and viro-immunological markers in HIV-1-infected patients

**DOI:** 10.1186/s12879-015-0768-4

**Published:** 2015-02-12

**Authors:** Katia Monteleone, Carla Selvaggi, Giulia Cacciotti, Francesca Falasca, Ivano Mezzaroma, Gabriella D’Ettorre, Ombretta Turriziani, Vincenzo Vullo, Guido Antonelli, Carolina Scagnolari

**Affiliations:** Pasteur Institute-Cenci Bolognetti Foundation, Department of Molecular Medicine, Laboratory of Virology, Sapienza University of Rome, Viale di Porta Tiburtina n 28, 00185 Rome, Italy; Department of Clinical Medicine, Sapienza University of Rome, Rome, Italy; Department of Public Health and Infectious Diseases, Sapienza University of Rome, Rome, Italy

**Keywords:** miRNA-29, HIV-1, IL-32, MxA

## Abstract

**Background:**

Several *in vitro* studies suggested the microRNA-29 (miRNA-29) family is involved in regulating HIV-1 and modulating the expression of interleukin (IL)-32, an anti-HIV-1 cytokine.

**Methods:**

To investigate the contribution of the miRNA-29 family to HIV-1 infection *in vivo*, we compared miRNA-29 expression in PBMC collected from 58 HIV-1-infected patients, naïve for antiretroviral therapy, and 21 gender- and age-matched HIV-1 seronegative healthy donors, using RT-Taqman assays. The relation between miRNA-29 levels and HIV-1 viro-immunological markers and the activation rate of antiviral immune response were also evaluated. In addition, we profiled miRNA-29 expression in CD4+ T lymphocytes and CD14+ monocytes collected from 5 antiretroviral treated HIV-1 infected patients.

**Results:**

miRNA-29b levels were higher in HIV-1-infected patients than in the control group (p < 0.001). There were no correlations with either HIV-1 RNA levels or CD4+ T count, whereas a significant correlation was found between miRNA-29-a/c levels and integrated HIV-1 DNA (miRNA-29a: p = 0.009, r = −0.448; miRNA-29c: p = 0.029; r = −0.381). When the HIV-1-infected patients were grouped on the basis of their plasma HIV-1 RNA and CD4+ T cell count, we also found that patients expressing the lowest levels of miRNA-29c showed high viraemia, low CD4+ T cell count and high levels of integrated HIV-1 DNA. Moreover, miRNA-29b levels were correlated with those of IL-32nonα (p = 0.028; r = −0.298). Patients expressing higher levels of miRNA-29b showed lower levels of MxA, an interferon-stimulated gene, also induced by IL-32 (p = 0.006 r = −0.397). Lastly, we found that CD4+ T lymphocytes and CD14+ monocytes shared similar miRNA-29a/b/c expression patterns but the amount of miRNA-29a/b/c, IL-32 isoforms and MxA were highly variable in these two cellular subsets.

**Conclusions:**

The miRNA-29 family could influence the clinical progression of HIV-1 infection, the HIV-1 proviral load and the innate immune response against HIV-1.

## Background

MicroRNAs (miRNAs) are small RNA molecules of ~22 nucleotides involved in the regulation of several biological processes [1]. Recently, miRNAs have also been shown to target and inhibit viral gene expression. Several interactions between HIV-1 and cellular miRNAs have been described, suggesting that altered miRNA profile expression could contribute to the pathogenesis of HIV-1 infection and HIV-1 latency in primary CD4+ T lymphocytes [2-5].

Among all the identified miRNAs, the miRNA-29 family has been suggested to play a pivotal role in regulating viral replication. The miRNA-29 family consists of four closely related members, miRNA-29a, miRNA-29b-1, miRNA-29b-2 and miRNA-29c, although the mature sequences of miRNA-29b-1 and miRNA-29b-2 are identical. They are characterized by the same “seed region” and are expressed in both T and B cells [6]. Recent reports showed that miRNA-29a downregulates nef expression interfering with HIV-1 replication in Jurkat cells [7,8]. Moreover it has been shown that miRNA-29a is highly expressed in human CD4+ T cells and is able to target the HIV-1 3'UTR region *in vitro* [9]. MiRNA-29a inhibition enhanced HIV-1 viral production and infectivity, whereas expression of miRNA-29a mimics suppressed viral replication [9]. In agreement with this observation, Sun et al. found that miRNA-29a/b, miRNA-155 and miRNA-21 levels were significantly reduced in HIV-1-infected CD4(+)CD8(−) peripheral blood mononuclear cells [10]. Furthermore, *ex vivo* experiments using HIV-1 infected lymphocytes reported a reduced expression of miRNA-29a, miRNA-29b, and miRNA-29c [10,11]. However, few studies have investigated the role of the miRNA-29 family in HIV-1 infection *in vivo*. Houzet and colleagues analyzed the expression of several miRNAs, including the miRNA-29 family, in PBMC from 36 HIV-1-infected individuals classified into four classes based on their CD4+ T cell counts and viral loads. They found miRNA profiles specific for those different classes of patients that plausibly correlate stage-specific miRNA alterations with the *in vivo* course of HIV-1 infection [12]. Moreover, Witwer and coworkers analysed miRNA profiles from healthy individuals, elite HIV-1 controllers, and untreated viraemic HIV-1 patients, also showing that HIV positive patients have an altered expression of the miRNA-29 family [13].

Interestingly, miRNA-29b was found to be involved in regulating the expression of interleukin (IL)-32, a cytokine with antiviral properties against HIV-1 [14-17], by directly targeting the IL-32 mRNA 3'-untranslated region [18], that can induce IFN-λ1, IFN-β and IFN-induced genes, such as MxA, PKR and APOBEC3G/3 F [17-20].

In order to investigate the contribution of the miRNA-29 family to HIV-1 regulation *in vivo*, we analysed miRNA-29a/b/c expression in HIV-1 positive patients naïve for antiretroviral therapy. Then we evaluated the influence of the miRNA-29 family on the main viro-immunological markers of HIV-1 infection by analysing the relationship between miRNA-29 family expression levels and plasma viraemia, CD4+ T cell count and levels of integrated HIV-1 DNA, which reflects the establishment of HIV-1 latency [21]. Moreover, to further characterize the relation between miRNA-29b and IL-32 expression, we investigated whether miRNA-29 levels influence the amount of IL-32 and MxA during HIV-1 infection. Finally, the miRNA-29a/b/c, IL-32 isoforms and MxA levels were measured in CD4+ T lymphocytes and CD14+ monocytes collected from antiretroviral treated HIV-1 patients with detectable viremia.

## Methods

### Study population

Peripheral blood samples obtained from 63 HIV-1-infected patients, both therapy-naïve (n = 58) and HAART-treated (n = 5), attending the Policlinico Umberto I University Hospital in Rome were included in this study. All patients were infected with the HIV-1 subtype B strain. No patients had any concurrent acute illness or infection including CMV disease, mycobacterium tubercolosisis, hepatitis B and C virus infections. The demographic and clinical characteristics of naïve and treated HIV-1-infected patients are reported in Table 1. Blood samples from 21 healthy gender- and age-matched individuals were also included in this study to compare the expression of miRNA-29a/b/c in naïve HIV-1 positive patients and healthy subjects.

**Table 1:** **Demographic and clinical characteristics of chronically HIV-1-infected patients and HIV seronegative healthy individuals**

**Item**	**Healthy individuals (n = 21) (A)**	**Naïve HIV-1-infected patients (n = 58) (B)**	**Treated HIV-1-infected patients (n = 5) (C)**	**A** ***vs*** **B**	**A** ***vs*** **C**
				***p*** **values**	***p*** **values**
Males, n (%)	13 (61.90)	43 (74.13)	4 (80)	0.549	0.809
Mean age ± SD (years)	42.38 ± 11.33	39.43 ± 11.84	44.25 ± 21.51	0.326	0.784
Virus subtype	NA	B	B	NA**	NA
HIV RNA (copies/ml)*	NA	34925 (143–1,405,000)	1446 (80–123,200)	NA	NA
CD4+ count (cells/mm3)*	NA	524 (22–1,200)	400 (350–895)	NA	NA
Time post infection (months)*	NA	12 (1–168)	84 (12–168)	NA	NA
Duration of HAART (years)*	NA	NA	6.5 (1–13)	NA	NA

*Data are expressed as median (range).

Differences in demographic characteristics between HIV-1-infected patients and HIV seronegative healthy individuals were evaluated using Student's t and Chi-squared tests.

**NA = not applicable.

The study was approved by the ethics committee of the Policlinico Umberto I Hospital, “Sapienza” University of Rome and informed consent was obtained from both HIV-1 positive patients and healthy individuals.

### Measurement of HIV-1 RNA and CD4+ T lymphocyte count

HIV-1 viral load was determined by versant HIV-1 RNA kPCR assay (Siemens Healthcare Diagnostic, Tarrytown NY, USA) which has a detection limit of 37 copies/ml. Absolute CD4+ T lymphocyte count was performed by FACScalibur flow cytometer (Becton Dickinson, San Jose, CA, USA).

### PBMC isolation

PBMC were isolated from naïve and treated HIV-1 infected patients and healthy individuals fresh blood by Ficoll-Hypaque density gradient centrifugation (Sigma-Aldrich, St. Louis, MO, USA) and dry pellets of 10^6^ PBMC were stored at −80°C.

### CD4+ T lymphocytes and CD14+ monocytes isolation

CD14+ monocytes and CD4+ T lymphocytes were isolated from PBMC collected from 5 treated HIV-1-infected patients by positive selection using the MACS® Technology (Miltenyi Biotec, Bergisch Gladbach, Germany), according to the manufacturer's protocol.

### TaqMan-based real time RT-PCR technique for microRNAs

MicroRNAs were quantified by real time RT-PCR Taqman assays (has-miRNA-29a, has-miRNA-29b, has-miRNA-29c, RNU6B, Applied Biosystems, Monza, Italy). Briefly, miRNAs were extracted from PBMCs, CD4+ T lymphocytes and CD14+ monocytes using the mirVana miRNA Isolation Kit (Ambion, Austin, TX, USA). Cellular miRNA purity was evaluated spectrophotometerically at the absorbance 230, 260 and 280 nm (Varioskan™ Flash Multimode Reader, Thermo Fisher Scientific Waltham, MA, USA). Then, miRNAs were reverse transcripted using TaqMan MicroRNA Reverse Transcription Kit, according to the manufacturer's protocols (Applied Biosystems); real time PCR was carried out in a final volume of 20 μl using LightCycler480 instrument (Roche, Basel, Switzerland). The constitutively expressed RNU6B was used as an internal control. Expression values of miRNA-29s were calculated by the comparative threshold cycle (Ct) method. In particular, the data were analyzed using the equation 2^−delta^*C*_T_, where Delta*C*_T_ = (*C*_T_ of target miRNA − *C*_T_ of internal control).

### TaqMan-based real time RT-PCR technique for mRNA expression evaluation

mRNA levels of IL-32α, IL-32nonα and MxA were assessed by real time RT-PCR using the LightCycler480 instrument, as previously described [17]. Briefly, total RNA was extracted from PBMC, CD4+ T lymphocytes and CD14+ monocytes using the TRIzol reagent (Invitrogen, Carlsbad, CA, USA), according to the manufacturer's protocol. The purity of RNA was evaluated spectrophotometerically at the absorbance 230, 260 and 280 nm (Varioskan™ Flash Multimode Reader, Thermo Fisher Scientific Waltham, MA, USA). Cellular RNA was reverse transcribed by using High-Capacity cDNA Archive Kit (Applied Biosystems) as previously specified [22]. Primers and probes for each gene were added to the Probes Master Mix (Roche, Basel, Switzerland) at 500 and 250 nM respectively, in a final volume of 20 μl. The housekeeping gene β-glucuronidase was used as an internal control. The primers and probe sequences used for β-glucuronidase gene were the following: Forward 5′-TCTGTCAAGGGCAGTAACCTG-3′, Reverse 5′-GCCCACGACTTTGTTTTCTG-3′, Probe 5′-(6FAM)TATGTCTTTCGATATGCAGCCAAGTTTTACCG3′(TAM)-3′. Gene expression values were calculated by the comparative Ct method. In particular, data were analyzed using the equation 2^−delta^*C*_T_, where Delta*C*_T_ = (*C*_T_ of target gene − *C*_T_ of housekeeping gene).

### TaqMan-based real time RT-PCR technique for HIV DNA measurement

Total DNA was extracted from PBMC collected from HIV-1 positive patients using the TRIzol reagent and then purified using QIAamp DNA Micro Kit (QIAGEN, Milan, Italy), according to the manufacturer's protocols. Integrated HIV DNA was quantified using two-step amplification. In the first step, HIV DNA primers designed to detect host-genome repetitive motifs (Alu) were paired with HIV-gag specific primers (30 cycle of amplification) to quantify integrated HIV DNA in patient samples as previously described [23]. In the second step a real time PCR for the LTR gene was performed using primers annealing in LTR gene [24]. Human telomerase reverse transcriptase (hTERT) was employed as a housekeeping gene and amplified in parallel with the HIV-1 gene. To quantify HIV-1 DNA we used a standard curve (fivefold dilutions of 8E5 cell DNA) and all samples from each patient were tested in the same assay. Results were expressed as number of HIV-1 DNA copies/10^6^ PBMC.

### Statistical analysis

Demographic characteristics of HIV-1 positive patients and healthy donors were compared using Student's t and Chi-squared tests.

Differences between HIV-1 positive patients and healthy donors in terms of miRNA-29 expression were analysed using the Mann–Whitney test. The same test was also used to compare miRNA-29 expression levels in HIV-1 positive patients divided into two classes on the basis of the their viral load (class I: HIV RNA >10000 copies/ml; class II: HIV RNA <10000 copies/ml) and to evaluate any difference in miRNA-29a/b/c levels and mRNA levels of MxA, IL-32α and IL-32nonα between CD4+ T lymphocytes and CD14+ monocytes.

Spearman's rho coefficient was calculated to assess the correlation between miRNA-29 levels and 1) the age of both patients and healthy individuals; 2) plasma viraemia and CD4+ T cell count; 3) integrated HIV DNA levels; 4) transcript levels of IL-32α, IL-32nonα and MxA.

Differences in miRNA-29 transcript levels in HIV positive patients stratified into five groups according to viral load and CD4+ T cell count (Table 2) were analysed using Kruskal-Wallis test. The same test was also used to evaluate any differences in expression among miRNA-29 s in HIV-1-infected individuals and healthy subjects, to compare miRNA-29 expression levels in HIV-1 positive patients divided into three groups on the basis of their CD4+ T cell count (low: <200 CD4+ T cells/mm^3^; intermediate: 200–500 CD4+ T cells/mm^3^; high: >500 CD4+ T cells/mm^3^) and to analyse differences in expression among miRNA-29a/b/c in CD4+ T lymphocytes and CD14+ monocytes.

A *p*-value less than 0.05 was considered statistically significant. All analysis were performed with SPSS v.17.0 for Windows.

**Table 2:** **Classifications of HIV-1-infected patients on the basis of CD4+ T cell count and viral load**

**Group**	**CD4+ T cell count (cells/mm** ^**3**^ **)**	**Plasma HIV-1 RNA (HIV RNA copies/ml)**	**Number of patients**
I	200-500	<10000	8
II	>500	<10000	6
III	<200	>10000	9
IV	200-500	>10000	11
V	>500	>10000	24

## Results

### Differential expression of miRNA-29 family members

**Figure 1:**
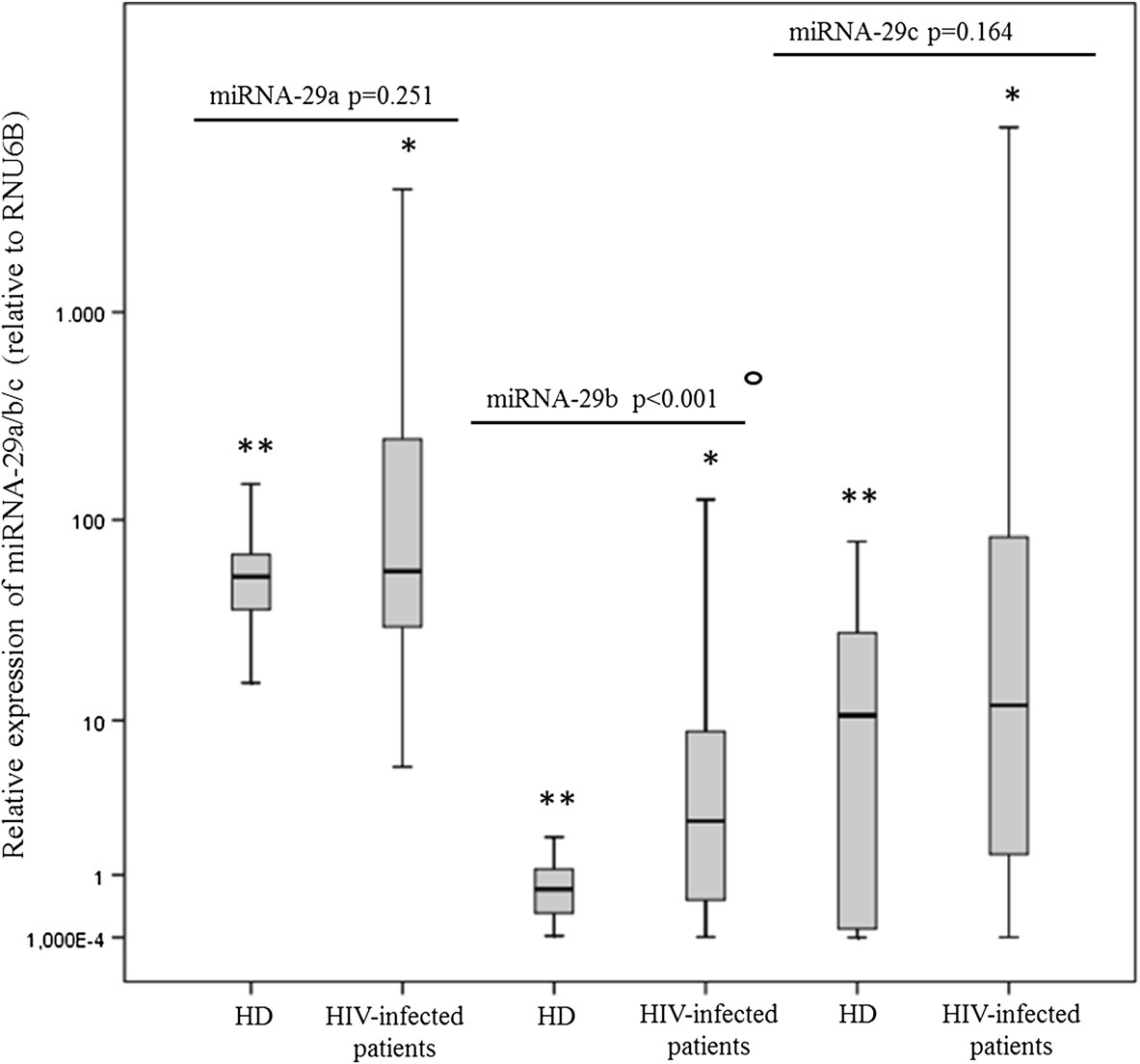
**Comparison of miRNA-29a/b/c levels in HIV-1-infected patients (n = 58) and gender- and age-matched healthy donors (HD) (n = 21).** MiRNA-29a/b/c levels were analysed in PBMC collected from HIV-1-infected patients and from healthy individuals using real time RT-PCR assays. MiRNA-29a/b/c levels are expressed as relative expression [ΔCt method] normalized to the levels of the constitutively expressed RNU6B. The relative quantity of miRNA-29a/b/c levels was calculated by the equation 2 − (ΔCt). Differences between HIV-1-infected patients and healthy donors in terms of miRNA-29 levels were analysed using the Mann–Whitney test. (°HIV-1-infected patients *vs* healthy donors: miRNA-29a, *p* = 0.251; miRNA-29b, *p* < 0.0001. miRNA-29c, *p =* 0.164). Kruskal-Wallis test was used to evaluate differences in expression among miRNA-29a/b/c in HIV-1-infected individuals and healthy subjects (*HIV-1-infected patients: *p* < 0.001; **healthy donors: *p* < 0.001).

We evaluated miRNA-29a, miRNA-29b and miRNA-29c levels in PBMC collected from 58 chronically HIV-1-infected patients naïve for antiretroviral treatment, and 21 gender- and age- matched HIV-seronegative healthy individuals. As expected, miRNA-29 transcript levels showed some degree of individual-to-individual variability both in untreated chronically HIV-1-infected patients and healthy donors [coefficient of variation: (HIV-infected patients = miRNA-29a: 309.97; miRNA-29b: 510.73; miRNA-29c: 320.08); (healthy donors = miRNA-29a: 57.82; miRNA-29b: 79.15; miRNA-29c: 98.87)] and a significant variation was observed among the levels of miRNA-29 members in both HIV-infected patients and healthy donors. In particular, we observed in both groups that miRNA-29a levels were higher than those of miRNA-29b/c, miRNA-29b levels were lower than those of miRNA-29a/c, whereas miRNA-29c levels showed intermediate levels of expression (HIV-infected patients: *p* < 0.001; healthy donors: *p* < 0.001) (Figure 1).

As shown in Figure 1, miRNA-29b levels were significantly higher in HIV-1-infected patients than those measured in the control group (*p* < 0.001). No significant difference was found in miRNA-29a and miRNA-29c levels between HIV-1-infected patients and healthy individuals, although we observed a trend toward higher levels of both miRNA-29 s in HIV-1-infected patients. The impact of age on miRNA-29 expression was strongly correlated between miRNA-29b levels and the age of healthy donors but not with that of HIV positive patients (*p* = 0.040; *r* = 0.574; *p* = 0.759; *r* = −0.042). In addition, we observed that gender was not associated with miRNA-29 levels in either group.

### miRNA correlations with plasma viral load or CD4+ T cells count

We analysed the relationship between miRNA-29 levels and those of well-known virological and immunological markers of HIV-1 infection: plasma viral load and CD4+ T cell count.

**Figure 2:**
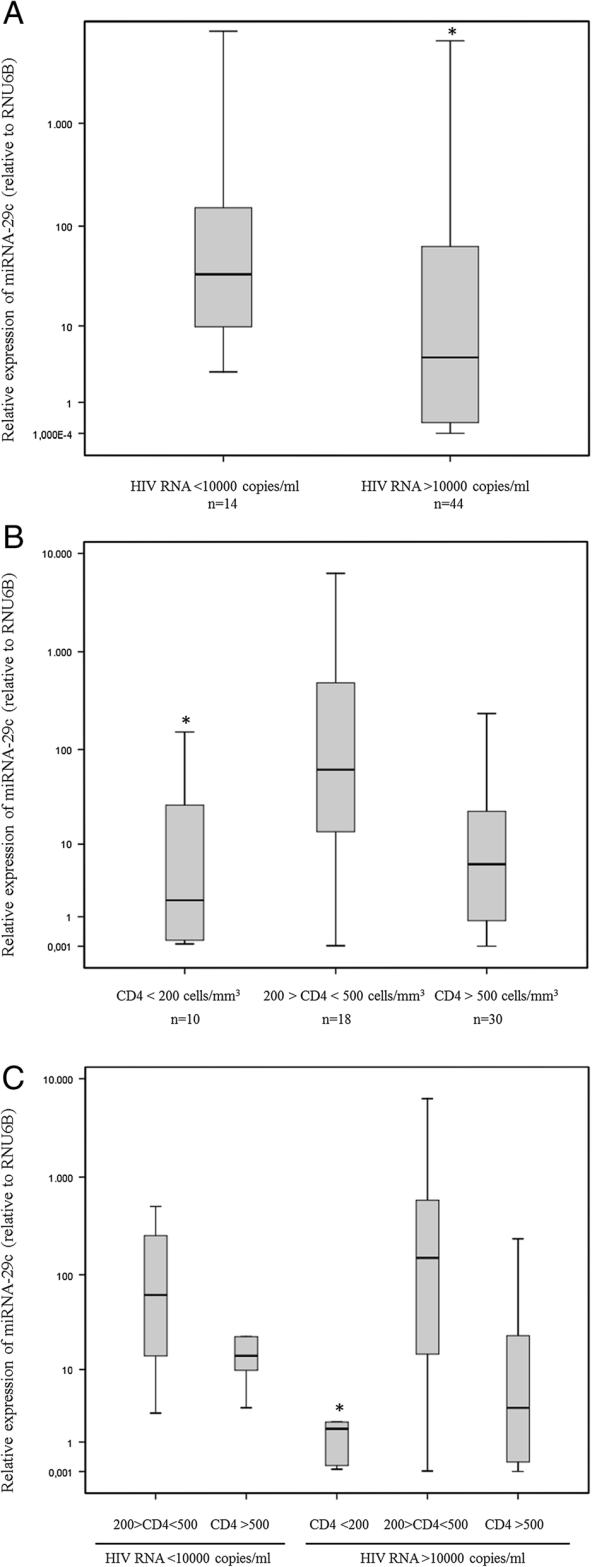
**Influence of miRNA-29c levels on viro-immunological markers of disease progression in HIV-1-infected patients (n = 58) naïve for antiretroviral therapy.** Panel **A**: miRNA-29c levels were compared in HIV-1 positive patients divided into two classes on the basis of their viral load (class I: HIV RNA >10000 copies/ml; class II: HIV RNA <10000 copies/ml). Data were analysed using the Mann–Whitney test (*p* = 0.038). Panel **B**: miRNA-29c levels were compared in HIV-1 positive patients divided into three groups on the basis of their CD4+ T cell count (low: <200 CD4+ T cells/mm^3^; intermediate: 200–500; CD4+ T cells/mm^3^; high: >500 CD4+ T cells/mm^3^). Data were analysed using the Kruskal-Wallis test (*p* = 0.019). Panel **C**: miRNA-29c levels were compared in HIV-1 positive patients divided into five groups on the basis of their viral load and CD4+ T cell count (Table 2). Data were analysed using the Kruskal-Wallis test (*p* = 0.019).

Results indicated that there were no correlations between miRNA-29a/b/c and HIV-1 RNA levels (Table 3), but when the HIV-1-infected patients were divided into two classes on the basis of the their plasma HIV-1 RNA (class I: >10000 copies/ml; class II: <10000 copies/ml), the patients expressing lower levels of miRNA-29c also had higher levels of viral load (Figure 2, Panel A) (*p* = 0.038).

**Table 3:** **Relation between miRNA-29a/b/c levels and viro-immunological parameters, IL-32 isoforms and MxA in 58 HIV-1-infected patients**

**Item**	**miRNA-29a**		**miRNA-29b**		**miRNA-29c**	
	*p*	*r*	*p*	*r*	*p*	*r*
HIV-1 RNA (copies/ml)	0.079	0.232	0.102	0.445	0.103	0.216
CD4+ T cell count (cells/mm^3^)	0.865	0.023	0.832	0.029	0.731	0.046
HIV-1 DNA (copies/10^6^ cells)	**0.009**	**−0.448**	0.176	−0.241	**0.029;**	**−0.381**
IL-32α	NA*	NA	0.318	−0.140	NA	NA
IL-32nonα	NA	NA	**0.028**	**−0.298**	NA	NA
MxA	NA	NA	**0.006**	**−0.397**	NA	NA

Spearman’s rho test; significant correlations are highlighted in bold.

*NA = not applicable.

Likewise, we failed to detect any significant association between miRNA-29 expression and CD4+ T count in our studied group (miRNA-29a: *p* = 0.865, *r* = 0.023; miRNA-29b: *p* = 0.832, *r* = 0.029; miRNA-29c: *p* = 0.731, *r* = 0.046) (Table 3) but when the chronically HIV-1-infected patients were grouped according to their CD4+ T cell count, the subset with <200 CD4+ T cells/mm^3^ had lower levels of miRNA-29c than the subsets with CD4+ T cell counts of between 200 and 500 and above 500 cells/mm^3^ (Figure 2, Panel B) (*p* = 0.019).

These data were also confirmed by a more detailed analysis based on the division of patients into five groups of HIV-1 seropositive individuals [i.e. patients with intermediate CD4+ T cell count and low viral load (group I), high CD4+ T cell count and low viral load (group II), low CD4+ T cell count and high viral load (group III), intermediate CD4+ T cell count and high viral load (group IV) and high CD4+ T cell count and high viral load (group V)] (Table 2). As expected, we found that each group was characterized by a different level of miRNA-29c expression (*p* = 0.019) and that again patients expressing the lowest levels of miRNA-29c showed high viraemia and low CD4+ T cell count (group III) (Figure 2, Panel C).

### MiRNA correlations with HIV-1 DNA

In an attempt to determine whether differentially expressed cellular miRNAs could influence HIV-1 proviral load, levels of miRNA-29a, miRNA-29b and miRNA-29c were examined for any significant correlation with levels of integrated HIV-1 DNA measured in PBMC collected from HIV-infected patients before starting antiretroviral therapy.

Our results indicated a significant negative correlation between miRNA-29a and miRNA-29c expression levels and those of integrated HIV-1 DNA (miRNA-29a: *p* = 0.009; *r* = −0.448, miRNA-29b: *p* = 0.176; *r* = −0.241, miRNA-29c: *p* = 0.029; *r* = −0.381) (Table 3).

### Expression of microRNA-29 a/b/c in CD14+ monocytes and CD4+ T lymphocytes

**Figure 3:**
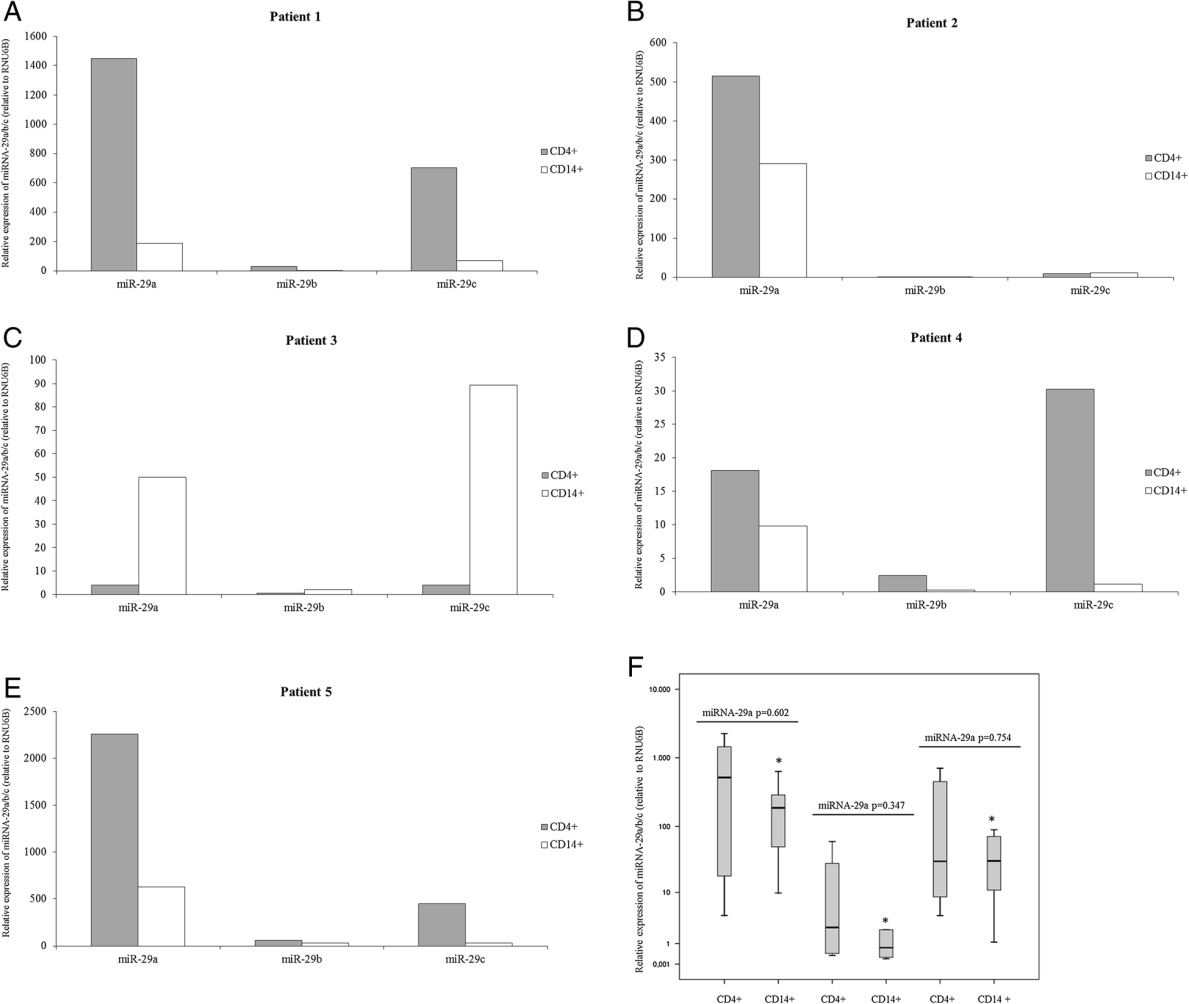
**Expression of miRNA-29a/b/c in CD14+ monocytes and CD4+ T lymphocytes collected from treated HIV-1-infected patients with detectable viremia (n = 5).** miRNA-29a/b/c levels were analysed in CD14+ monocytes and CD4+ T lymphocytes using real time RT-PCR assays. Panel **A-E**. Patient 1: viral load = 146 HIV RNA copies/ml; CD4+ T cell count = 450 cells/mm^3^. Patient 2: viral load = 80 HIV RNA copies/ml; CD4+ T cell count = 350 cells/mm^3^. Patient 3: viral load = 3278 HIV RNA copies/ml; CD4+ T cell count = 340 cells/mm^3^. Patient 4: viral load = 123,200 HIV RNA copies/ml; CD4+ T cell count = 400 cells/mm^3^. Patient 5: viral load = 1446 HIV RNA copies/ml; CD4+ T cell count = 895 cells/mm^3^. Panel **F**. Differences between CD14+ monocytes and CD4+ T lymphocytes in terms of miRNA-29 levels collected from treated HIV-1-infected patients were analysed using the Mann–Whitney test (CD4+ T lymphocytes *vs* CD14+ monocytes: miRNA-29a, p = 0.602; miRNA-29b, p = 0.347; miRNA-29c, p = 0.754). Kruskal-Wallis test was used to evaluate differences in expression among miRNA-29a/b/c in HIV-1-infected individuals and healthy subjects (CD4+ T lymphocytes: p = 0.114; *CD14+ monocytes: p = 0.021).

In an attempt to evaluate whether distinct cellular subsets harbored unique miRNA profiles, the miRNA-29a/b/c levels were measured in CD4+ T lymphocytes and CD14+ monocytes collected from HIV-1 infected patients who didn’t achieve a virological suppression in response to antiretroviral therapy*.* As previously observed in PBMC of naïve HIV patients, results confirmed that miRNA-29a levels were higher than those of miRNA-29b/c, miRNA-29b levels were lower than those of miRNA-29a/c, whereas miRNA-29c levels showed intermediate levels of expression in both CD4+ T lymphocytes and CD14+ monocytes collected from treated HIV-1 patients (Figure 3). However, the differences in terms of miRNA-29a/b/c expression reached statistical significance only when CD14+ monocytes were analyzed (Figure 3, CD4+ T lymphocytes: p = 0.114; CD14+ monocytes: p = 0.021). Moreover, we found that some HIV-1 patients expressed higher levels of miRNA29a-c in CD4 + T lymphocytes compared to those in CD14+ monocytes while others manifested an opposite miRNA29a-c pattern in these two cellular subsets (Figure 3). Due to the high inter-patients variability of miRNA-29a/b/c expression, the median values of miRNA-29 a, b, and c were not different between CD4+ T lymphocytes and CD14+ monocytes (Figure 3, CD4+ lymphocytes *vs* CD14+ monocytes: miRNA-29a, p = 0.602; miRNA-29b, p = 0.347; miRNA-29c, p = 0.754).

### *In vivo* relation between miRNA-29b and IL-32

To investigate whether the transcript levels of miRNA-29b affect IL-32 expression and, in turn, the antiretroviral response, we measured IL-32 (α and nonα isoforms) mRNA levels in PBMC collected from 58 HIV-1-infected patients naïve for antiretroviral treatment. Interestingly, we found an inverse weak correlation between miRNA-29b and IL-32nonα levels in HIV positive patients (*p* = 0.028; *r* = −0.298) whereas no significant correlation was found with IL-32α levels (Table 3).

**Figure 4:**
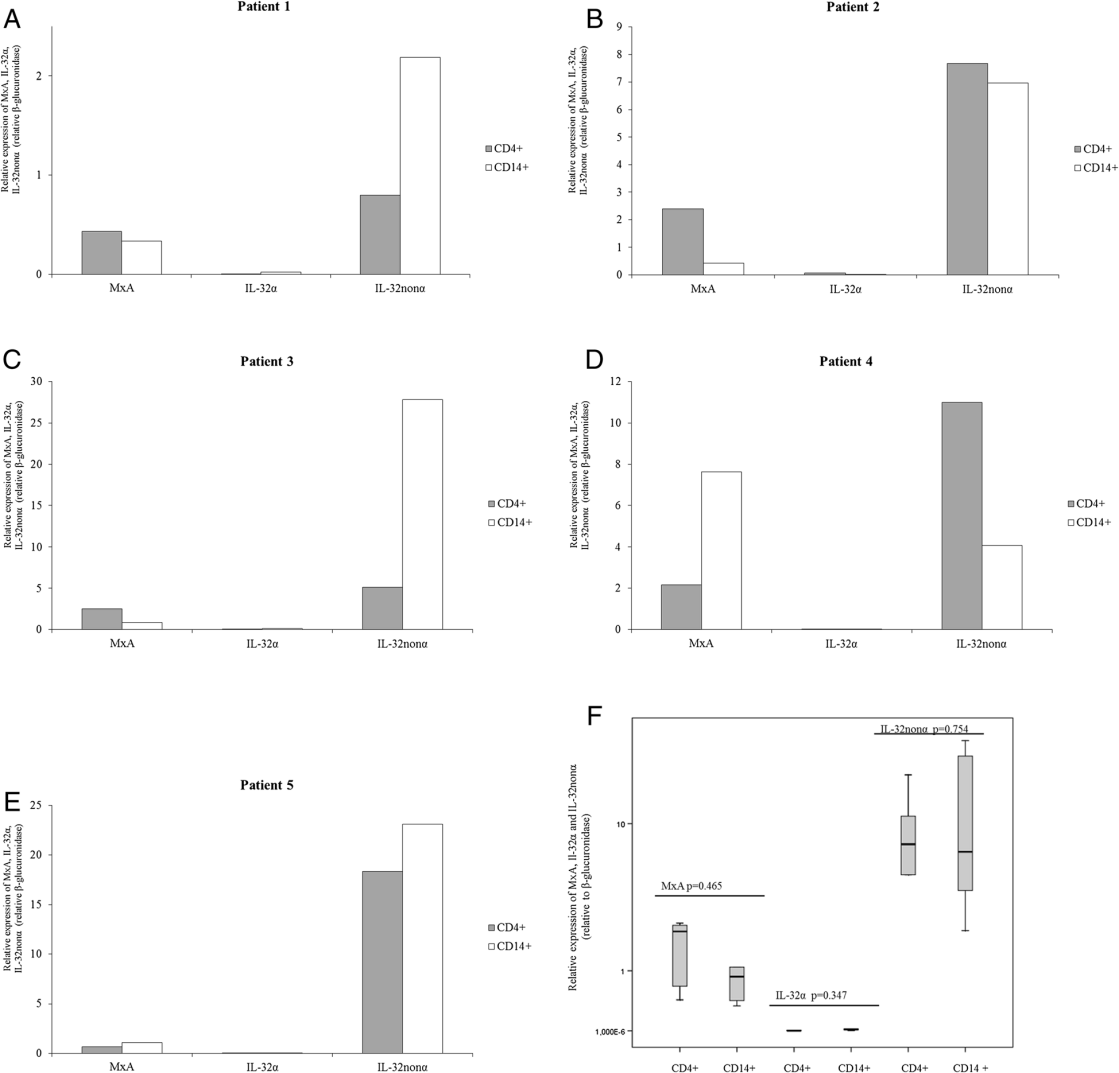
**mRNA expression of MxA, IL-32α and IL-32nonα in CD14+ monocytes and CD4+ T lymphocytes collected from treated HIV-1-infected patients with detectable viremia (n = 5).** mRNA levels of MxA, IL-32α and IL-32nonα were analysed using real time RT-PCR assays. mRNA levels are expressed as relative expression [ΔCt method] normalized to the levels of the constitutively expressed β-glucuronidase gene. Panel **A-E**. Patient 1: viral load = 146 HIV RNA copies/ml; CD4+ T cell count = 450 cells/mm^3^. Patient 2: viral load = 80 HIV RNA copies/ml; CD4+ T cell count = 350 cells/mm^3^. Patient 3: viral load = 3278 HIV RNA copies/ml; CD4+ T cell count = 340 cells/mm^3^. Patient 4: viral load = 123,200 HIV RNA copies/ml; CD4+ T cell count = 400 cells/mm^3^. Patient 5: viral load = 1446 HIV RNA copies/ml; CD4+ T cell count = 895 cells/mm^3^. Panel **F**. Differences in mRNA levels between CD14+ monocytes and CD4+ T lymphocytes collected from treated HIV-1-infected patients (n = 5) were analysed using the Mann–Whitney test (CD4+ T lymphocytes *vs* CD14+ monocytes: MxA, p = 0.465; IL-32α, p = 0.347; IL-32nonα, p = 0.754).

Furthermore we evaluate whether miRNA-29b levels influence the transcript levels of MxA, a well-established type I and III IFN-stimulated gene, which is also induced by IL-32. We found a strong positive correlation between IL-32nonα and MxA transcript levels (*p* < 0.001; *r* = 0.593) and patients expressing higher levels of miRNA-29b showed lower levels of MxA (*p* = 0.006 *r* = −0.397) (Table 3). Then, we tried to establish which cellular subset, CD4+ T lymphocytes and CD14+ monocytes, could be responsible of the relationships observed between miRNA-29s, IL-32 and MxA in PBMC of naïve HIV-1 patients. As previously observed for miRNA-29a/b/c levels, the expression of IL-32 isoforms (α and nonα) and MxA exhibited a high degree of variability in CD4+ T lymphocytes and CD14+ monocytes and they were not different in these cellular subsets (Figure 4, CD4+ lymphocytes *vs* CD14+ monocytes: MxA, p = 0.465; IL-32α, p = 0.347; IL-32nonα, p = 0.754).

## Discussion and conclusions

Recent reports have indicated the miRNA-29 family as a putative regulator of key processes against HIV-1 infection. Thus, we compared the expression profile of miRNA-29a, miRNA-29b and miRNA-29c in HIV-1-infected patients and healthy donors, and evaluated whether and how differences in expression could influence the clinical progression of HIV-1 infection and the antiviral immune response.

Interestingly, we found a significant positive correlation between miRNA-29b levels and the age of healthy donors but not with that of HIV positive patients, suggesting an age-dependent regulation of miRNA-29b expression in healthy individuals. In agreement with this observation, Zhang et al. reported that mechanisms of transcriptional regulation of miRNA transcripts and altered expression of Argonaut proteins contribute to age-related changes in the expression of some miRNA and miRNA* strands [25]. The loss of correlation between age and miRNA-29b levels observed in the HIV-infected patients could be explained as a consequence of viral pathogenesis: HIV-1 infection *in vivo* is expected to exert physiologic effects on T-cell function which could be reflected in significant miRNA changes. Directly related to this, we found that levels of miRNA-29b expression were significantly higher in HIV-1-infected patients compared to the control group. No significant correlation was found between miRNA-29a and miRNA-29c expression levels and the age of either healthy donors or HIV-1 positive patients. We also failed to detect any significant difference between miRNA-29a and miRNA-29c transcript levels measured in healthy donors and HIV-1 positive patients, although we did observe a trend toward higher levels of both miRNAs in HIV-1-infected patients. This could be explained by the fact that although the three mature members of the miRNA-29 family share a common seed region sequence and are predicted to target largely overlapping sets of genes, they exhibit differential regulation in several cases and different subcellular distribution [26]. In addition, their stable expression levels are likely to depend on expression of the two clusters, alternative splicing of primary RNA and differential decay, all factors which may vary in a cell-specific manner [6].

In line with our results, it was recently reported that HIV-1-infected patients are characterized by an altered miRNA expression profile compared to healthy donors. Witwer and coworkers described an altered PBMC miRNA profile in elite suppressors and untreated viraemic patients compared to uninfected controls. However, they found that among miRNAs with significant expression changes expression levels were more often lower in viraemic individuals. For example, control PBMC had higher mean levels than viraemic PBMC of all miRNA-29 family members [13]. Furthermore, Houzet and colleagues profiled miRNA expression in PBMC from 36 HIV-1 seropositive individuals categorized into four classes based on their CD4+ T cell counts and viral loads. They found that specific miRNA signatures, including miRNA-29s, can be observed for each class [12]. Having observed that HIV-1 infection is associated with altered patterns of miRNA-29 expression, we tried to evaluate whether this phenomenon would affect the plasma viral load and CD4+ T cell count. In a first analysis, we did not find any significant association between miRNA-29 expression levels and plasma HIV RNA levels. This observation is in agreement with Witwer et al., who found no correlations with viral load in the viraemic group, although miRNA-29a has been reported to silence HIV-1 *in vitro* [7-9]. We also failed to find any significant association between miRNA-29s expression and CD4+ T cell count, whereas Witwer et al.’s study found both negative and positive correlations between several miRNAs, including miRNA-29a, and CD4+ T cell counts. However, this correlation was only present when elite suppressor, not included in our study population, and viraemic patients were grouped together. To further analyze the relationship between miRNA-29s expression and the viro-immunological markers of HIV-1 infection, we grouped the chronically HIV-1-infected patients on the basis of their plasma HIV RNA and CD4+ T cell count, to represent different stages of the infection. Although there was no significant difference in expression levels of miRNA-29a and miRNA-29b between patients with more or less severe HIV-1 infection, interestingly we found that miRNA-29c levels were related to both viral load and CD4+ T cell count. Indeed, patients expressing lower levels of miRNA-29c also had higher levels of viraemia and lower levels of CD4+ T cell count, suggesting a strong relation between miRNA-29c and these clinical markers of HIV-1 infection. This relation may imply that baseline lower levels of miRNA-29c expression in some individuals could negatively influence the progression of HIV-1 infection. Alternatively, the presence of HIV-1 or the host response against HIV-1 may reduce miRNA-29c expression, by inducing CD4+ T-cell depletion.

Several studies reported that the onset of HIV-1 latency could be influenced by cellular miRNA [27], but the role of miRNA-29s in controlling this phenomenon has not been well characterized. For the first time to our knowledge, our results show that miRNA-29c expression levels are significantly and negatively correlated with levels of integrated HIV-1 DNA. Thus, in our study population, patients with lower levels of miRNA-29c not only showed higher levels of plasma viraemia and a lower CD4+ T cell count, but also had higher levels of HIV-1 DNA, confirming the association between low levels of expression of miRNA-29c and poor prognosis. Probably, the higher rate of proliferation observed in patients with a lower CD4+ T cell count led to a larger overall reservoir size. On the other hand, we cannot exclude that by suppressing HIV-1 production miRNA-29a could regulate viral gene expression, modulating the viral life cycle and promoting the onset and maintenance of latency. Moreover, given that we found the same significant trend in miRNA-29a levels, miRNA-29a and miRNA-29c could influence HIV-1 latency by a common mechanism. However, miRNA-29a-c and HIV-1 DNA integrated levels were quantified only in total PBMCs, and may have missed differences in various cell types, such as resting central memory T cells and translational memory T cells, which serve as major sites for HIV-1 latency [28].

Interestingly, in this study we also found that miRNA-29a/b/c signature was similar in both CD4+ T lymphocytes and CD14+ monocytes to that previously observed in total PBMC of naive HIV-1 infected patients. Furthermore, we observed that the amount of miRNA-29a-c measured in CD4+ T lymphocytes and CD14+ monocytes were highly variable and not different in these cellular subsets. These findings indicate that both CD4+ T lymphocytes and CD14+ monocytes can actively contribute to the production of miRNA-29 a/b/c during HIV-1 infection and suggest that CD4+ T cell alteration does not explain all differential expression of miRNA-29a/b/c recorded in HIV-1 patients. In agreement, Witwer KW et al. reported that some cellular miRNAs that have been found at high levels across PBMC subsets or are even enriched in CD4+ T-cell subsets [29]-and would therefore be expected to decline along with CD4+ T cells were, to the contrary, negatively correlated with CD4+ T cell count [13]. Further studies are needed to characterize the effects of HIV-1 on miRNA-29s expression in PBMC and specific cell types both in vivo and, as far as possible, in primary culture ex vivo.

Our study is also the first to evaluate the influence of miRNA-29b on the expression of different isoforms of a novel anti-HIV cytokine, namely IL-32, during chronic HIV-1 infection. Given that six isoforms of IL-32 have been identified, we chose to detect IL-32α and the other IL-32 isoforms (β, γ, δ, ε, ζ) separately, using a real time PCR assay previously developed in our laboratory [17]. We found a weak inverse correlation between miRNA-29b and IL-32nonα levels in PBMC collected from HIV-1-infected patients, whereas no significant correlation was found with IL-32α levels, suggesting that miRNA-29b specifically targets other isoforms of IL-32. Our data are in agreement with those reported by Li and coworkers, who showed that miRNA-29b and IL-32 mRNA levels were negatively correlated in PBMC samples from HBV-infected patients [18]. However, further analyses will be needed to confirm the presence of these relationships with a larger sample size of HIV-1 infected patients and to identify which specific isoform/isoforms of IL-32 is/are regulated by miRNA-29b.

One of the tools used by IL-32 to fight viruses, including HIV-1, is its ability to induce MxA expression [17]. We previously demonstrated that IL-32γ can significantly induce expression of IFN stimulating genes (ISGs), including MxA, in PBMC collected from healthy donors, although to a lower extent than IFNα2b [17]. Moreover, recent reports showed that IL-32 exerts its antiviral activity through the induction of IFN-λ1 and IFN-β expression, which in turn act by inducing ISGs [18,19]. Thus, we evaluated the influence of miR-29b expression on the transcript levels of MxA in HIV-infected patients. As expected, patients expressing higher levels of miRNA-29b showed lower levels of MxA, confirming that MxA induction is regulated by IL-32, both directly and through a type I and III IFN-mediated pathway. Interestingly, we also found that CD4+ T lymphocytes as well as CD14+ monocytes were capable of producing miRNA-29b, IL-32 isoforms and MxA indicating that these cellular subsets were involved in the activation of the IL-32- and IFN-mediated antiviral response during HIV-1 infection. Taken together, these data suggest that the up-regulation of miRNA-29b observed in HIV-infected patients has a strong negative impact on the antiviral immune response and that the virus may take advantage of the change in the normal cell miRNA profile. On the other side, considering the complex and to some extent controversial role played by IFN-α/β in HIV-1 disease [30], our results indicated that miRNA-29b would contribute to the regulation of the rate of IFN activation by suppressing the IL-32 nonα isoforms levels during HIV-1 infection. However, several cellular pathways are regulated by both miRNA-29 and IFN subtypes highlighting the complexity of phenomenon analyzed [31-33]. In this regards, miRNA-29 can regulate and activate T-box transcription factors and IFN-γ production in helper T cells [31]. Furthermore, epigenetic changes mediated by miRNA-29 can induce IFN-λ1 production during viral infection and the suppression of the IFN-α receptor expression can be mediated by miRNA-29 in thymic epithelium to increase the threshold for infection-associated thymic involution [32,33].

In conclusion, we showed that transcription levels of all mature members of the miRNA-29 family are highly variable in HIV-1-infected patients and that the miRNA-29b expression pattern is altered in this population compared to healthy individuals. We also found that miRNA-29c expression is closely correlated with markers of HIV-1 clinical outcome, such as plasma viral load and CD4+ T cell count, and that both miRNA-29a and miRNA-29c could affect the HIV-1 proviral load. In addition, we demonstrated that miRNA-29b levels influence the rate of IL-32nonα and MxA expression, highlighting the role of the miRNA-29 family as a double-edged sword during *in vivo* HIV-1 infection.

## References

[1] He L, Hannon GJ (2004). MicroRNAs: small RNAs with a big role in gene regulation. Nat Rev Genet.

[2] Triboulet R, Mari B, Lin YL, Chable-Bessia C, Bennasser Y, Lebrigand K (2007). Suppression of microRNA-silencing pathway by HIV-1 during virus replication. Science.

[3] Zhou R, Rana TM (2013). RNA-based mechanisms regulating host-virus interactions. Immunol Rev.

[4] Yeung ML, Bennasser Y, Myers TG, Jiang G, Benkirane M, Jeang KT (2005). Changes in microRNA expression profiles in HIV-1-transfected human cells. Retrovirology.

[5] Huang J, Wang F, Argyris E, Chen K, Liang Z, Tian H (2007). Cellular microRNAs contribute to HIV-1 latency in resting primary CD4+ T lymphocytes. Nat Med.

[6] Liston A, Papadopoulou AS, Danso-Abeam D, Dooley J (2012). MicroRNA-29 in the adaptive immune system: setting the threshold. Cell Mol Life Sci.

[7] Hariharan M, Scaria V, Pillai B, Brahmachari SK (2005). Targets for human encoded microRNAs in HIV genes. Biochem Biophys Res Commun.

[8] Ahluwalia JK, Khan SZ, Soni K, Rawat P, Gupta A, Hariharan M (2008). Human cellular microRNA hsa-miR-29a interferes with viral nef protein expression and HIV-1 replication. Retrovirology.

[9] Nathans R, Chu CY, Serquina AK, Lu CC, Cao H, Rana TM (2009). Cellular microRNA and P bodies modulate host-HIV-1 interactions. Mol Cell.

[10] Sun G, Li H, Wu X, Covarrubias M, Scherer L, Meinking K (2012). Interplay between HIV-1 infection and host microRNAs. Nucleic Acids Res.

[11] Hayes AM, Qian S, Yu L, Boris-Lawrie K (2011). Tat RNA silencing suppressor activity contributes to perturbation of lymphocyte miRNA by HIV-1. Retrovirology.

[12] Houzet L, Yeung ML, de Lame V, Desai D, Smith SM, Jeang KT (2008). MicroRNA profile changes in human immunodeficiency virus type 1 (HIV-1) seropositive individuals. Retrovirology.

[13] Witwer KW, Watson AK, Blankson JN, Clements JE (2012). Relationships of PBMC microRNA expression, plasma viral load, and CD4+ T-cell count in HIV-1-infected elite suppressors and viremic patients. Retrovirology.

[14] Nold MF, Nold-Petry CA, Pott GB, Zepp JA, Saavedra MT, Kim SH (2008). Endogenous IL-32 controls cytokine and HIV-1 production. J Immunol.

[15] Rasool ST, Tang H, Wu J, Li W, Mukhtar MM, Zhang J (2008). Increased level of IL-32 during human immunodeficiency virus infection suppresses HIV replication. Immunol Lett.

[16] Smith AJ, Toledo CM, Wietgrefe SW, Duan L, Schacker TW, Reilly CS (2011). The immunosuppressive role of IL-32 in lymphatic tissue during HIV-1 infection. J Immunol.

[17] Monteleone K, Di Maio P, Cacciotti G, Falasca F, Fraulo M, Falciano M (2014). Interleukin-32 isoforms: expression, interaction with interferon-regulated genes and clinical significance in chronically HIV-1-infected patients. Med Microbiol Immunol.

[18] Li Y, Xie J, Xu X, Liu L, Wan Y, Liu Y (2013). Inducible interleukin 32 (IL-32) exerts extensive antiviral function via selective stimulation of interferon λ1 (IFN-λ1). J Biol Chem.

[19] Nakayama M, Niki Y, Kawasaki T, Takeda Y, Ikegami H, Toyama Y (2013). IL-32-PAR2 axis is an innate immunity sensor providing alternative signaling for LPS-TRIF axis. Sci Rep.

[20] Zepp JA, Nold-Petry CA, Dinarello CA, Nold MF (2011). Protection from RNA and DNA viruses by IL-32. J Immunol.

[21] Ruelas DS, Greene WC (2013). An integrated overview of HIV-1 latency. Cell.

[22] Scagnolari C, Midulla F, Pierangeli A, Moretti C, Bonci E, Berardi R (2009). Gene expression of nucleic acid-sensing pattern recognition receptors in children hospitalized for respiratory syncytial virus-associated acute bronchiolitis. Clin Vaccine Immunol.

[23] Liszewski MK, Jianqing J, O’Doherty U (2009). Detecting HIV-1 integration by repetitive-sampling Alu-gag PCR. Methods.

[24] Viard JP, Burgard M, Hubert JB, Aaron L, Rabian C, Pertuiset N (2004). Impact of 5 years of maximally successful highly active antiretroviral therapy on CD4 cell count and HIV-1 DNA level. AIDS.

[25] Zhang X, Azhar G, Wei JY (2012). The expression of microRNA and microRNA clusters in the aging heart. PLoS One.

[26] Kriegel AJ, Liu Y, Fang Y, Ding X, Liang M (2012). The miR-29 family: genomics, cell biology, and relevance to renal and cardiovascular injury. Physiol Genomics.

[27] Swaminathan G, Navas-Martín S, Martín-García J (2014). MicroRNAs and HIV-1 infection: antiviral activities and beyond. J Mol Biol.

[28] Van Lint C, Bouchat S, Marcello A (2013). HIV-1 transcription and latency: an update. Retrovirology.

[29] Rossi RL, Rossetti G, Wenandy L, Curti S, Ripamonti A, Bonnal RJ (2011). Distinct microRNA signatures in human lymphocyte subsets and enforcement of the naive state in CD4+ T cells by the microRNA miR-125b. Nat Immunol.

[30] Hughes R, Towers G, Noursadeghi M (2012). Innate immune interferon responses to human immunodeficiency virus-1 infection. Rev Med Virol.

[31] Steiner DF, Thomas MF, Hu JK, Yang Z, Babiarz JE, Allen CD (2011). MicroRNA-29 regulates T-box transcription factors and interferon-γ production in helper T cells. Immunity.

[32] Fang J, Hao Q, Liu L, Li Y, Wu J, Huo X (2012). Epigenetic changes mediated by microRNA miR29 activate cyclooxygenase 2 and lambda-1 interferon production during viral infection. J Virol.

[33] Papadopoulou AS, Dooley J, Linterman MA, Pierson W, Ucar O, Kyewski B (2011). The thymic epithelial microRNA network elevates the threshold for infection-associated thymic involution via miR-29a mediated suppression of the IFN-α receptor. Nat Immunol.

